# MRI and PET Alterations in Adult Skull Base Tumors: A Narrative Review of Proton Versus Photon Radiotherapy

**DOI:** 10.3390/diagnostics16081166

**Published:** 2026-04-14

**Authors:** Gokoulakrichenane Loganadane, Valentin Calugaru, Dimitri Anzellini, Benjamin Nicaise, Sarah Mezghani, Nam P. Nguyen, Brandi R. Page

**Affiliations:** 1 Department of Radiation Oncology, Institut Curie, 75005 Paris, France; valentin.calugaru@curie.fr (V.C.); benjamin.nicaise@curie.fr (B.N.); 2 Department of Radiation Oncology, Sant’Andrea Hospital, Sapienza University, 00189 Rome, Italy; 3 Department of Radiology, Institut Curie, 75005 Paris, France; 4 Department of Radiation Oncology, Howard University, Washington, DC 20060, USA; namphong.nguyen@yahoo.com; 5 Department of Radiation Oncology, Johns Hopkins University School of Medicine, Baltimore, MD 21205, USA; brandi.page@gmail.com

**Keywords:** skull base tumor, radiation-induced brain injury (RIBI), radiation-induced contrast enhancement (RICE), proton therapy, relative biological effectiveness, radiation necrosis

## Abstract

**Background:** Radiotherapy is essential for skull base tumor management but carries the risk of radiation-induced brain injury (RIBI). This spectrum ranges from transient radiation-induced contrast enhancement (RICE) to irreversible necrosis. Distinguishing these entities from tumor progression is critical, particularly with the increasing adoption of proton therapy. **Methods:** A comprehensive narrative review of the peer-reviewed literature was conducted up to October 1, 2025. The search strategy focused on adult patients treated for skull base malignancies, synthesizing data on dose–volume metrics, incidence rates, and modality-specific toxicity profiles. **Results:** RIBI represents a pathophysiological continuum. (a) Descriptive imaging patterns: In prospective proton therapy series, focal RICE occured in 15% of patients, typically at a median of 12 months, and often resolved spontaneously. (b) Modality comparison: Although proton therapy reduces integral brain dose versus photon therapy, elevated linear energy transfer (LET) at the distal Bragg peak may contribute to focal radiation-associated image changes (RAIC), particularly in the temporal lobes. (c) Risk stratification and diagnosis: Risk increased when >1% of the healthy brain received >57.6 Gy (Relative Biological Energy (RBE)) or when V67Gy exceeded 0.17 cc. Advanced MRI and amino acid positron emission tomography (PET) improved differentiation between radiation effects and tumor recurrence. **Conclusions:** Post-radiation imaging changes are common and often benign. Distinguishing RICE from progression requires multimodal imaging and adherence to specific dose constraints. Management should prioritize surveillance for asymptomatic lesions.

## 1. Introduction

The skull base is one of the most complex anatomical regions of the human body, forming the floor of the cranial cavity and the roof of the aerodigestive tract [1]. It includes the clivus, jugular foramen, temporal bone, petrous apex, and sellar and parasellar regions, and is traversed by critical neurovascular structures such as the cranial nerves, internal carotid arteries, brainstem, and optic apparatus [2]. Tumors in this confined region challenge local control and functional preservation due to proximity to eloquent neural structures. They include aggressive bone malignancies (chordomas, chondrosarcomas), compressive benign or borderline lesions (meningiomas, pituitary adenomas, craniopharyngiomas), and invasive epithelial cancers such as adenoid cystic carcinoma (ACC) and undifferentiated carcinoma of nasopharyngeal type (UCNT), which often spread perineurally to the cavernous sinus and Meckel’s cave. Despite their rarity, their cumulative morbidity necessitates highly conformal radiotherapy to achieve tumor control while protecting adjacent brain structures.

Radiotherapy (RT) plays a central role in management, either as definitive treatment for unresectable disease or adjuvant therapy after subtotal resection. Given the high risk of toxicity in this region, technological evolution has aimed to maximize the therapeutic ratio [3]. Modern photon techniques—including stereotactic radiosurgery (SRS), intensity-modulated radiotherapy (IMRT), and volumetric-modulated arc therapy (VMAT) achieve high conformality through computer-optimized beam modulation [4]. However, photons deposit an unavoidable exit dose, resulting in low-to-intermediate integral dose to surrounding brain structures, potentially contributing to late toxicity.

Proton-based therapy (PBT) exploits the Bragg peak, depositing maximal energy at a defined depth with minimal exit dose [5,6]. This significantly reduces integral brain dose and improves sparing of organs at risk (OARs) such as the cochlea, hippocampus, and temporal lobes. For radioresistant tumors such as chordomas requiring 70–74 Gy (RBE), PBT enables dose escalation that may be unsafe with photons [7]. Carbon-ion therapy offers higher relative biological effectiveness (RBE) but may increase necrosis risk. Despite technological advances, radiation-induced brain injury remains a serious complication. Skull base osteoradionecrosis is a rare but potentially devastating late effect characterized by hypovascularity, hypoxia, and bone necrosis, potentially leading to cranial neuropathies, meningitis, or carotid hemorrhage [8]. High prescribed doses and re-irradiation common in recurrent meningiomas or pituitary adenomas, further increase risk. Accurate interpretation of post-treatment MRI is therefore essential.

Clival tumors are surrounded by the medial temporal lobes and brainstem, while jugular foramen lesions involve the cerebellum and lower cranial nerves. ACC commonly spreads perineurally to the cavernous sinus and Meckel’s cave. Delivering tumoricidal doses while sparing optic pathways and temporal lobes demands meticulous planning, as proton range uncertainties and anatomical variations may result in unintended dose to critical structures [9,10]. For example, resolution of edema or sinus aeration changes may alter tissue density, shifting the Bragg peak and overdosing adjacent neural structures.

Distinguishing radiation-induced changes from tumor progression—particularly in the re-irradiation setting—remains a major neuro-oncologic challenge. Multiparametric MRI, amino acid PET imaging (^18^F-FET, ^11^C-MET), and emerging radiomics approaches improve diagnostic accuracy and guide retreatment decisions [11].

Proton range uncertainties are influenced by weight loss, sinus filling, and tissue density variations. Reduced water-equivalent path length (WEPL) may cause distal overshoot into structures such as the brainstem [12]. Additionally, RBE is not biologically constant at 1.1; experimental data demonstrate higher RBE values at the distal edge of the Bragg peak due to increased linear energy transfer (LET) [13]. Clinically, this may produce a 1–3 mm biological range extension, potentially contributing to endothelial injury and radiation-induced contrast enhancement (RICE), even when physical dose constraints appear acceptable [14]. Sinusitis or variable sinus aeration may similarly alter proton range: increased density shortens range, risking target underdosage, whereas aeration allows deeper penetration [15]. Modern pencil beam scanning (PBS) incorporates robust optimization (typically ±3.5%) to mitigate such uncertainties [16]. Nevertheless, adaptive imaging, periodic quality assurance computed tomography (CT), and replanning capability remain essential, particularly in patients with weight changes, anatomical variability, or hardware [17].

This narrative review was conducted to address three practical questions: (1) What are the expected MRI/PET patterns and their temporal evolution after adult skull base radiotherapy? (2) How do incidence estimates, dose–volume predictors, and clinical risk factors compare between proton and photon techniques? (3) Which advanced MRI sequences and PET tracers best support differentiation between treatment effect and tumor progression? To answer these, we summarize MRI alterations in adult patients with skull base tumors treated with proton and photon radiotherapy, outline underlying pathophysiology and clinical risk factors, compare modality-specific outcomes, and highlight advanced MRI and PET techniques for differentiating radiation injury from tumor recurrence. This synthesis is needed because post-treatment imaging changes are increasingly encountered with the expanding use of proton therapy, yet terminology remains heterogeneous and misinterpretation may prompt unnecessary interventions. Emphasis is placed on chordoma, chondrosarcoma, meningioma, adenoid cystic carcinoma, pituitary adenoma, craniopharyngioma, UCNT, and germinoma in the adult population.

## 2. Materials and Methods

### 2.1. Literature Search Strategy

A comprehensive narrative review was executed to identify and synthesize the peer-reviewed literature published from database inception up to 1 October 2025. Overall, the included studies spanned publication years 1998–2025. The primary electronic databases queried included PubMed, Scopus, and Web of Science. The search strategy employed a combination of medical subject headings (MeSH) and free-text keywords related to the anatomical site (“skull base tumor,” “clivus,” and “cavernous sinus”), pathology (“chordoma,” “chondrosarcoma,” “meningioma,” “adenoid cystic carcinoma,” and “pituitary adenoma”), therapeutic modality (“proton therapy,” “photon therapy,” “intensity-modulated radiotherapy,” and “stereotactic radiosurgery”), and adverse events (“radiation-induced brain injury,” “radiation necrosis,” “pseudoprogression,” “radiation-induced contrast enhancement,” and “contrast-enhancing brain lesion”).

### 2.2. Study Selection and Inclusion Criteria

To ensure the clinical relevance and homogeneity of the review, eligible studies were required to meet the following criteria:

1. Target population: Adult patients (aged ≥18 years) treated for benign or malignant tumors of the skull base. Pediatric cohorts were explicitly excluded due to the developing brain’s distinct radiosensitivity, differing neurocognitive endpoints, and unique survivorship considerations.
2. Intervention: Treatment involving external beam radiotherapy, specifically focusing on the comparison and characterization of outcomes following proton beam therapy (PBT) versus photon-based techniques (IMRT, VMAT, and SRS).
3. Outcomes of interest: Studies reporting quantitative data on the incidence, temporal evolution, or dosimetric predictors of radiation-induced imaging changes (e.g., RICE, RAIC, and necrosis). Additionally, studies evaluating the diagnostic performance of advanced imaging modalities (MRI sequences and PET tracers) in distinguishing radiation effects from tumor recurrence were prioritized. Exclusion criteria included pediatric-only cohorts (aged under 18 years), preclinical studies, and publications without post-radiotherapy neuroimaging outcomes.

### 2.3. Data Synthesis and Quality Assessment

Priority was given to data derived from prospective clinical trials, large retrospective institutional registries, and systematic reviews. Case reports and small case series were included only when they provided unique insights into novel pathophysiological mechanisms or rare clinical entities. Reference lists of selected primary articles were manually screened (“snowball sampling”) to identify additional relevant studies that may have been missed by the initial electronic search. Data were synthesized qualitatively to describe the spectrum of injury, with quantitative extraction of dose–volume constraints (e.g., V60Gy, Dmax) where reported in the literature.

## 3. Results

### 3.1. Terminology and Definitions

The literature describing post-radiation imaging changes is characterized by significant heterogeneity in nomenclature. To ensure clarity and consistency, this review adopts the harmonized definitions proposed by the European Particle Therapy Network (EPTN), summarized in Table 1 [9].

Radiation-induced brain injury (RIBI): Used herein as the comprehensive umbrella term encompassing all parenchymal brain changes attributable to radiotherapy. This spectrum ranges from mild, reversible inflammatory changes to severe, irreversible tissue death.

Radiation-induced contrast enhancement (RICE): Defines the appearance of focal, distinct contrast-enhancing lesions in the irradiated brain parenchyma that were not present on pre-treatment imaging. These lesions are typically transient, often asymptomatic, and frequently resolve without therapeutic intervention. In this review, RICE is the preferred term for focal enhancing nodules described in proton therapy cohorts [18,19].

Radiation-associated image change (RAIC): Frequently used in the head and neck cancer literature, this term is largely synonymous with RICE but is occasionally used more broadly to include associated T2/FLAIR signal abnormalities (edema/gliosis) independent of enhancement [20]. For the purposes of quantitative dose constraints, RICE and RAIC are treated as functionally equivalent entities representing focal BBB disruption.

Pseudoprogression (PsP): Refers to a sub-type of early delayed injury characterized by an increase in contrast enhancement and edema within the tumor bed or surgical cavity that mimics tumor progression but subsequently stabilizes or regresses. While classically described in glioblastoma patients receiving concurrent temozolomide (occurring within 3–6 months), the term is distinct from the late-onset RICE seen in the healthy brain tissue of skull base patients.

Radiation necrosis (RN): Represents the severe, irreversible end of the injury spectrum. It is characterized histologically by coagulative necrosis and radiologically by significant mass effect, “soap-bubble” or “Swiss-cheese” enhancement, and persistent perilesional edema. Unlike RICE, necrosis often requires surgical or medical intervention.

### 3.2. Pathophysiology of Radiation-Induced Brain Injury

The pathogenesis of RIBI is a complex, multifactorial process involving vascular, glial, and immunological components. The primary event is widely considered to be radiation-induced damage to the microvasculature (Table 2). High-dose radiation triggers apoptosis in endothelial cells, leading to blood–brain barrier (BBB) compromise [21]. This disruption allows the extravasation of serum proteins and contrast agents, manifesting radiologically as RICE or RAIC.

Simultaneously, radiation depletes the population of oligodendrocyte type-2 astrocyte (O-2A) progenitor cells, impairing the remyelination capacity of the white matter. This demyelination contributes to the T2/FLAIR hyperintensities observed in early delayed injury. In the chronic phase, a cycle of hypoxia and expression of vascular endothelial growth factor (VEGF) drives aberrant neovascularization. These fragile, leaky vessels further exacerbate edema and enhancement. If the inflammatory and hypoxic insult exceeds the tissue’s repair capacity, coagulative necrosis ensues, characterized by the classic “radiation necrosis” seen in late delayed injury.

### 3.3. Categories of Injury

Radiation-induced brain injury can be categorized by timing and imaging features:

#### 3.3.1. Acute Injury (Within Hours to Days)

Rare in high-precision skull base radiotherapy because the brain receives fractionated doses. When present, it reflects blood–brain barrier disruption with vasogenic edema and T2/FLAIR hyperintensity. It usually resolves spontaneously and seldom causes lasting deficits [22].

#### 3.3.2. Early Delayed Injury (1–6 Months)

Characterized by demyelination and transient oligodendrocyte damage. MRI shows diffuse T2/FLAIR hyperintensity in white matter with minimal or patchy contrast enhancement; involvement of deep temporal lobes is typical after skull base irradiation. Pseudoprogression, defined as transient new or enlarged enhancing lesions within months of therapy, belongs to this category. It occurs in 20–30% of patients, particularly when concurrent chemotherapy (e.g., temozolomide) is used [23]. Pseudoprogression tends to peak around three months and resolves over subsequent months.

#### 3.3.3. Late Delayed Injury (>6 Months)

Includes RICE and radiation necrosis. RICE encompasses new contrast-enhancing lesions that may or may not have clinical symptoms; lesions often appear as small nodular enhancements or rim-like patterns in the temporal or frontal lobes and may improve or resolve spontaneously [18]. Radiation necrosis represents irreversible parenchymal destruction with cavitation and “soap-bubble” or ring enhancement [24]. Necrosis most commonly manifests 12–36 months after radiotherapy but may occur several years later. In contemporary practice, severity is generally graded using the Common Terminology Criteria for Adverse Events (CTCAE), distinguishing asymptomatic radiographic findings (Grade 1) from symptomatic edema requiring medical therapy (Grade ≥ 2) and severe necrosis necessitating surgical intervention (Grade 3–4) [18,19].

### 3.4. MRI Alterations in Skull Base Tumors

Radiotherapy for skull base tumors can induce a spectrum of changes in healthy brain tissue, ranging from transient inflammation to irreversible tissue damage. Distinguishing these treatment effects from tumor recurrence is a critical challenge in post-treatment surveillance (Table 3).

#### 3.4.1. Pseudoprogression

Pseudoprogression represents forms of early or intermediate radiation injury that mimic tumor progression [23]. Radiographically, these present as new or enlarging contrast-enhancing lesions accompanied by T2/FLAIR hyperintensity and mass effect. While pseudoprogression is well-characterized in high-grade glioma populations, as reported in 31% (32/103) of newly diagnosed glioblastoma patients treated with RT plus temozolomide at the first post-treatment MRI, the rates in adult skull base tumors are not well established. Incidence may differ substantially outside of temozolomide-based regimens; therefore, glioblastoma-derived estimates should be generalized to skull base tumors with caution.

#### 3.4.2. Radiation-Associated Image Changes (RAIC)

RAIC serve as a broad umbrella term describing the spectrum of morphological and signal alterations observed on MRI following radiotherapy. Unlike specific diagnoses like necrosis, RAIC encompasses a wider array of radiographic abnormalities, including localized T2/FLAIR white matter hyperintensities which may appear with or without contrast enhancement. These changes reflect an early-to-intermediate neuroinflammatory response or transient edema. Risk is primarily driven by dose–volume parameters. In a mixed head-and-neck cohort receiving at least 40 Gy_RBE to the brain, RAIC occurred in 17.3%, with a 3-year cumulative incidence of 14.3%. Most cases were asymptomatic. High focal brain doses were a strong predictor; specifically, a volume receiving 67 Gy_RBE greater than 0.17 cc (V67Gy > 0.17 cc) significantly predicted the development of RAIC [20].

#### 3.4.3. Radiation-Induced Contrast Enhancement (RICE)

RICE is a specific subset of imaging changes characterized by focal blood–brain barrier disruption in irradiated tissue, most commonly affecting the temporal lobes or brainstem. In a prospective analysis of 421 patients treated with pencil-beam scanning PBT for CNS and skull base tumors, the cumulative incidence of RICE was 15% (63/421) at a median follow-up of 24 months. The vast majority of these cases remained asymptomatic. Events were classified as CTCAE grade 1 in 10.5%, grade 2 in 3.1%, and grade 3 in 1.4%. Symptomatic RICE (defined as grade ≥ 2) specifically accounted for just 4.5% of the total cohort, with previous in-field irradiation identified as the only significant predictor for developing symptoms [18]. Timeline: For patients who developed RICE, the median onset was approximately 11.8 months, and among resolving lesions, the median duration was 9.0 months.

#### 3.4.4. Radiation Necrosis

Radiation necrosis is the most severe late effect, representing irreversible tissue destruction. MRI typically reveals ring-enhancing lesions with central necrosis and profound surrounding edema. Risk Factors: Necrosis risk is linked to larger irradiated volumes (V60–70), higher maximum doses (Dmax), and older age [25]. Differentiating necrosis from tumor recurrence is difficult as both exhibit contrast enhancement. Incidence by Modality: Photon Therapy: Classic 2D series reported temporal lobe necrosis rates of 2–10% in nasopharyngeal and head-and-neck cancers. IMRT reduces this risk to approximately 2–5%. Particle Therapy: Proton therapy generally lowers high-dose volumes, potentially reducing necrosis rates compared to photons or carbon ions. A 2019 review emphasized that protons have a lower necrosis incidence (17%) compared to carbon ions (64%) and a shorter latency period [26]. However, in a study of skull base chordoma and chondrosarcoma treated with proton or carbon therapy, 21.5% experienced temporal lobe reactions and 13.9% developed frank necrosis at a median onset of 20 months [25]. Another PBT series identified temporal lobe injury in 10.4% of patients receiving 63–74 Gy_RBE [27]. Clinical Management: Manifestations depend on location; temporal lobe injury may cause seizures (temporal lobe epilepsy), memory impairment, or psychomotor slowing [28], while lesions near the cavernous sinus may cause cranial nerve deficits. Management strategies include corticosteroids, anti-epileptics, bevacizumab, hyperbaric oxygen, and surgical resection for severe cases.

#### 3.4.5. Contrast-Enhancing Brain Lesions (CEBL) and RIBI Spectrum

The term CEBL is increasingly used to capture the heterogeneous group of lesions that present with contrast enhancement on MRI after radiotherapy. This classification includes pseudoprogression, RICE, necrosis, and small vascular malformations. As imaging sensitivity and survivorship improve, the recognition of subtle CEBL is becoming more common. These lesions can appear years after therapy and may exhibit a waxing and waning course. In contemporary practice, radiation-induced brain injury is typically graded using the Common Terminology Criteria for Adverse Events (CTCAE), which standardizes toxicity reporting according to symptom severity, functional limitation, and the requirement for therapeutic intervention [18]. For research purposes, RIBI is often graded by size, symptoms, and the necessity of intervention.

### 3.5. Tumor-Specific Considerations and Incidence

#### 3.5.1. Glioblastoma vs. Skull Base Tumors: Differentiating Pseudoprogression

The incidence of pseudoprogression is heavily influenced by tumor biology and concurrent systemic therapy. In the landmark prospective analysis by Brandes et al., pseudoprogression was reported in 31% (32/103) of glioblastoma patients treated with photon radiotherapy and concurrent temozolomide [23]. Crucially, this phenomenon was strongly correlated with MGMT promoter methylation status (occurring in 91% of methylated patients vs. 41% of unmethylated patients) [29]. This high incidence is driven by the synergistic inflammatory effect of chemotherapy and radiation on a biologically aggressive tumor. Therefore, these rates should not be generalized to adult skull base tumors (e.g., chordoma, meningioma), which are typically lower-grade, are treated without concurrent temozolomide, and exhibit different radiobiological responses.

#### 3.5.2. Chordoma and Chondrosarcoma

For skull base chordomas and chondrosarcomas, which require ultra-high radiation doses (often 70–74 Gy RBE), the primary concern is late temporal lobe injury rather than early pseudoprogression. A 2024 retrospective analysis by Mattke et al. evaluating patients treated with particle radiotherapy for skull base chordoma and chondrosarcoma provides critical insight into these late toxicity patterns [25]. Overall, 21.5% of the patient cohort developed temporal lobe reactions, which encompassed a mix of both symptomatic and asymptomatic radiographic changes. Of these, 13.9% progressed to true, irreversible radiation necrosis. These changes are distinctly late-onset; the median time to development of temporal lobe reactions was 20 months post-radiotherapy, necessitating rigorous and extended MRI follow-up. Chondrosarcomas generally exhibit a more favorable overall prognosis and are often treated with marginally lower total radiation doses (e.g., 68–70 GyRBE) compared to clival chordomas, which often receive maximum dose escalation (72–74 GyRBE). Because temporal lobe toxicity is so heavily dependent on the V60–70 threshold, this slight reduction in target dose translates to a measurable difference in toxicity. As a result, the rate of temporal lobe reactions is observed to be marginally lower in chondrosarcoma subgroups, sitting at approximately 18%, compared to the higher incidence seen in chordoma patients [25].

#### 3.5.3. Head and Neck Cancers (Nasopharyngeal/Adenoid Cystic Carcinoma)

In patients with head and neck malignancies extending to the skull base, the temporal lobes are frequently incidental targets. Engeseth et al. reported a crude incidence of RAIC in 17.3% of patients treated with proton therapy [20]. Notably, 100% of the RAIC lesions in this cohort were asymptomatic and detected solely on surveillance MRI. The 3-year actuarial rate of developing RAIC was 14.3%. This underscores that while imaging changes are common, clinical toxicity is relatively rare in the modern proton era, provided that dose constraints are respected.

#### 3.5.4. Meningioma

Data specific to proton-treated meningiomas suggest a favorable toxicity profile, though pseudoprogression can occur. A study comparing proton vs. photon therapy for meningiomas found that while T2/FLAIR changes were common in both groups, proton therapy was associated with a higher rate of transient contrast enhancement changes (RICE), likely attributable to the LET effects at the beam end-range [9]. However, severe adverse events remain uncommon.

#### 3.5.5. Pituitary Adenoma and Craniopharyngioma

Pituitary adenomas and craniopharyngiomas lie close to the optic apparatus and hypothalamus. Stereotactic photon radiotherapy or proton therapy is used when surgery is incomplete. Radiation-induced injury may affect adjacent temporal lobes, optic tracts, or hypothalamus. Pseudoprogression may occur in the optic chiasm or hypothalamic region and must be distinguished from tumor enlargement [18]. Because these tumors typically receive lower doses than chordomas (~45–54 Gy), significant necrosis is rare; however, re-irradiation increases risk.

#### 3.5.6. Germinoma and Other Germ Cell Tumors

Intracranial germinomas often involve the suprasellar or pineal region. While proton therapy is increasingly used, adult germinoma data on radiation injury remain limited because of high cure rates and relatively low doses (36–45 Gy). Pseudoprogression can occur after chemoradiation but usually resolves.

### 3.6. Dosimetric Predictors and Quantitative Constraints

#### 3.6.1. Photon Versus Proton Therapy

In photon therapy, toxicity is often driven by the low-to-intermediate-dose exposure.

While proton therapy significantly lowers the integral dose to normal tissues, toxicity risk may instead be influenced by elevated LET, especially in high-LET regions near the distal fall-off [30,31]. At the very end of the proton range (the distal edge of the Bragg peak), the LET rises sharply. This results in an increased RBE [32]. While clinical planning systems assume a constant RBE of 1.1, the biological reality at the distal edge may be an RBE of 1.3 to 1.6 or higher [33]. If this distal edge falls within the vascular-rich temporal lobe, it can cause focal endothelial damage (RICE) even if the physical dose appears safe.

Photon therapy remains widely used. IMRT reduces temporal lobe doses compared with older techniques but still exposes larger volumes to intermediate doses. Nasopharyngeal carcinoma series using photons report temporal lobe necrosis rates of 2–5%, with risk increasing for total dose > 70 Gy and concurrent chemotherapy. Re-irradiation or brachytherapy further elevates risk. In chordoma patients, photon therapy may require large margins because of proximity to the brainstem, leading to more frequent necrosis. Consequently, proton therapy is considered standard for many skull base tumors.

#### 3.6.2. Validated Dose Constraints

Quantitative analysis of dosimetric parameters has yielded specific thresholds that predict the development of RICE and RAIC (Table 3). These constraints are essential for optimizing treatment plans to minimize the risk of late toxicity. Table 3 provides a summary mapping these estimates to their source cohorts.

The “Hot Spot” Constraint (V67Gy ≥ 0.17 cc): In the analysis by Engeseth et al., the volume of the brain receiving ≥ 67 Gy(RBE) (V67Gy) was the strongest predictor of RAIC [20]. Specifically, when the V67Gy exceeded just 0.17 cc (a minute volume representing a focal “hot spot”), the probability of developing RAIC surged to 63%. This finding emphasizes that even microscopic volumes of high-dose exposure in the temporal lobe can precipitate focal BBB breakdown.

The Healthy CNS Threshold (D1% > 57.6 Gy): Lütgendorf-Caucig et al. identified a broader volume constraint in a prospective proton cohort [18]. They found that when 1% of the healthy CNS volume (D1%) received a dose greater than 57.6 Gy (RBE), the hazard ratio for developing RICE increased significantly (HR 3.73). This metric serves as a useful surrogate for the overall burden of high-dose radiation on the brain parenchyma.

#### 3.6.3. Clinical Risk Factors

Clinical factors contribute to susceptibility [34]. Older age correlates with reduced vascular repair and increased necrosis [35]. Diabetes and hypertension contribute to microvascular injury and may heighten risk [36]. Previous cranial irradiation predisposes to necrosis; in the PBT study, prior in-field radiation significantly increased symptomatic RICE [18]. Tumor location influences injury pattern; clival tumors risk temporal lobe injury, whereas jugular foramen tumors may affect the cerebellum (Table 4).

### 3.7. Advanced Imaging Techniques

Standard MRI sequences (T1-weighted, T2/FLAIR, and gadolinium-enhanced T1) provide excellent anatomical detail but may not reliably distinguish radiation injury from tumor recurrence. Advanced MRI techniques offer quantitative biomarkers.

#### 3.7.1. Diffusion-Weighted Imaging (DWI)

DWI interrogates the cellular density of the lesion. Tumor recurrence, characterized by tightly packed, dividing cells, typically restricts water diffusion, leading to high signal on trace images and low values on Apparent Diffusion Coefficient (ADC) maps. Conversely, radiation necrosis is hypocellular, consisting of liquefactive necrosis and edema, which facilitates water movement [37]. Consequently, necrosis typically exhibits elevated ADC values. However, the overlap is significant, particularly in mixed lesions or pseudoprogression, where inflammatory infiltrates can mimic cellularity.

#### 3.7.2. Perfusion-Weighted Imaging (PWI)

PWI, specifically Dynamic Susceptibility Contrast (DSC), measures cerebral blood volume (CBV). Tumors induce angiogenesis, leading to elevated relative CBV (rCBV) with rapid contrast uptake and washout. Radiation necrosis, driven by vascular occlusion and endothelial apoptosis, is generally hypoperfused with low rCBV. A threshold ratio of rCBV < 0.6–0.7 is often cited as suggestive of necrosis, while ratios > 1.75 strongly favor recurrence. Dynamic Contrast-Enhanced (DCE) MRI, which quantifies vascular permeability (Ktrans), can also be useful, as radiation injury often results in “leaky” vessels with high permeability but low blood volume [38].

#### 3.7.3. Magnetic Resonance Spectroscopy (MRS)

MRS assesses metabolic profiles. Recurrent tumor typically exhibits high choline peaks and reduced N-acetylaspartate (NAA), whereas necrosis shows lipid and lactate peaks with low choline. In skull base tumors, MRS can be challenging due to bone–air interfaces but remains valuable when feasible [39].

#### 3.7.4. Radiomics and Artificial Intelligence

Radiomics extracts quantitative features (texture, shape, intensity) from MRI. Machine-learning models integrating radiomic features and clinical data have shown good performance in differentiating necrosis from recurrence. Although data are limited for skull base tumors, radiomics could improve risk stratification and guide follow-up intervals [40].

#### 3.7.5. PET Imaging

PET provides metabolic information complementary to MRI. 18F-FDG PET measures glucose metabolism; recurrent tumor generally demonstrates high uptake, whereas necrosis shows low uptake due to tissue death. However, high baseline brain glucose uptake in the temporal lobe reduces specificity. Amino acid PET tracers such as 18F-fluoroethyl-L-tyrosine (18F-FET), 11C-methionine (11C-MET), and 18F-fluoro-L-dihydroxyphenylalanine (18F-FDOPA) offer better lesion-to-background contrast because normal brain has low amino acid uptake. Studies in gliomas indicate that FET-PET differentiates recurrence from radiation necrosis with sensitivity >80% [41]. In skull base tumors, FET- or MET-PET helps evaluate lesions adjacent to the cortex or skull base and may identify early pseudoprogression. 18F-FMISO PET assesses hypoxia and may predict necrosis risk by identifying hypoxic areas susceptible to radiation injury. Combining PET and MRI (PET/MRI) allows simultaneous structural and metabolic assessment and has potential to guide adaptive radiotherapy [42]. More prospective studies are needed.

18F-FET has a half-life of 110 min and is widely available compared with 11C tracers that require an on-site cyclotron. High lesion-to-background ratios (often >2) allow detection of small lesions. 18F-FDOPA, originally developed for movement disorders, accumulates in catecholamine-producing tissues and has shown utility for head-and-neck tumors. In adenoid cystic carcinoma and nasopharyngeal carcinoma, FDOPA-PET may reveal perineural spread beyond the field of MRI. 18F-FMISO and other nitroimidazole tracers accumulate in hypoxic cells; hypoxia predisposes to radioresistance and may predict areas at risk for necrosis when irradiated [40]. Preliminary studies suggest that high FMISO uptake before radiotherapy correlates with subsequent necrosis, although data for skull base tumors are limited. Hybrid PET/MRI scanners offer co-registration and reduced examination time, enabling longitudinal monitoring of metabolic changes alongside structural MRI. As PET tracers evolve (e.g., 18F-fluciclovine), their role in skull base tumor imaging will likely expand.

#### 3.7.6. Evolution of Lesions over Time

**Figure 1:**
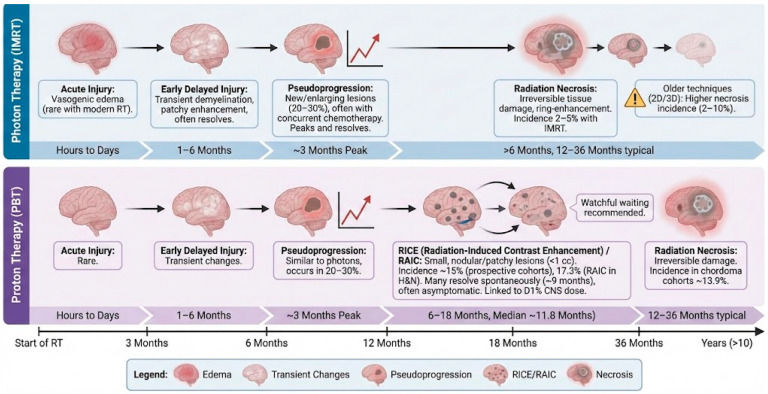
Temporal evolution and radiographic characteristics of radiation-induced brain injury (RIBI) following photon versus proton therapy.

**Figure 2:**
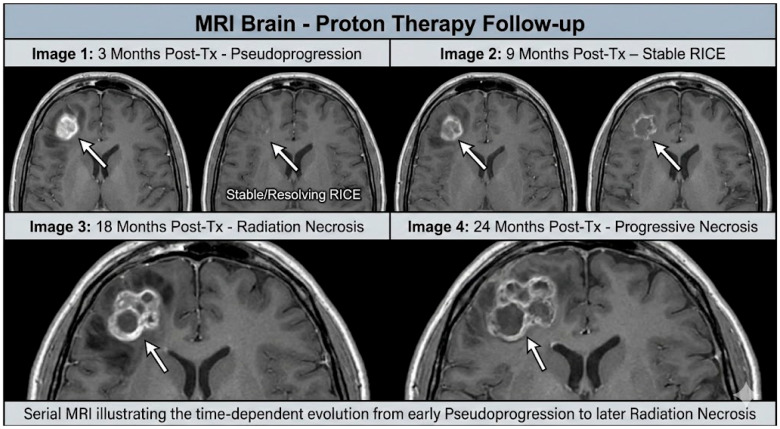
Longitudinal MRI evaluation of RICE, pseudoprogression, and radiation necrosis following proton therapy. RICE: Radiation-induced contrast enhancement.

Radiation-induced lesions evolve dynamically (Figure 1). Pseudoprogression typically peaks within three months of therapy and resolves by six months [43]. RICE lesions may appear as small nodules or patchy enhancement at 6–18 months and often resolve by 12–24 months [40]. Radiation necrosis usually manifests 1–3 years post-therapy but can develop as late as 10 years; lesions may expand and then stabilize or regress. In proton cohorts, many RICE lesions improved or resolved spontaneously, highlighting the importance of watchful waiting [20]. However, symptomatic necrosis may progress, requiring intervention (Figure 2).

### 3.8. Clinical Management

Management depends on severity and symptoms. Observation is appropriate for small, asymptomatic RICE lesions because many regress. Corticosteroids reduce edema but have systemic side effects; they are used in the short term for symptomatic pseudoprogression or RICE. Anti-angiogenic therapy with bevacizumab has been shown to reduce symptoms and radiological lesions by decreasing VEGF-mediated permeability. Hyperbaric oxygen and pentoxifylline with vitamin E have anecdotal benefits. Boswellia serrata extract, particularly its active component acetyl-11-keto-β-boswellic acid (AKBA), shows preliminary evidence for reducing cerebral edema associated with brain radiotherapy, but there is insufficient evidence to support its use specifically for treating established radiation necrosis. Relevant clinical evidence comes from a small prospective, randomized, double-blind, placebo-controlled trial of 44 patients with primary or secondary malignant cerebral tumors receiving radiotherapy. Patients received either Boswellia serrata 4200 mg/day or placebo during radiation treatment. A reduction in brain edema of >75% was found in 60% of patients receiving BS compared to 26% receiving placebo (*p* = 0.023), measured by T2-weighted MRI immediately after radiotherapy completion [44].

Surgical resection is reserved for life-threatening mass effect or when diagnosis is uncertain. The potential of proton therapy to decrease necrosis supports its use for re-irradiation when feasible.

### 3.9. Future Directions

Despite advances, several gaps remain. Standardized terminology for radiation-induced lesions (e.g., pseudoprogression, RICE, and CEBL) is needed to facilitate comparisons. Prospective registries capturing detailed dose–volume metrics, imaging findings, and clinical outcomes across tumor types and modalities will clarify risk factors. Integration of advanced MRI, PET, and radiomics into surveillance protocols could enable earlier detection and personalized follow-up intervals. New systemic therapies (e.g., immune checkpoint inhibitors) may interact with radiation, altering pseudoprogression incidence; understanding these interactions in skull base tumors warrants investigation. Finally, exploring the role of carbon-ion therapy with improved dose painting techniques may reduce necrosis while maintaining high control rates [45].

**Table 1:** Glossary of key terms in radiation-induced brain injury.

Term	Acronym	Definition	Typical Onset After RT	Clinical Context/Notes
Radiation-Induced Brain Injury	RIBI	Umbrella term encompassing the full spectrum of radiation-related effects on normal brain parenchyma, including reversible imaging changes and irreversible necrosis.	Variable (months to years)	General category; includes RICE/RAIC, pseudoprogression, and radiation necrosis.
Radiation-Induced Contrast Enhancement	RICE	Focal nodular or patchy contrast-enhancing lesions within irradiated brain tissue, often asymptomatic and potentially reversible.	6–18 months (median ≈ 12 months)	Frequently reported in proton therapy series; often self-limited.
Radiation-Associated Image Changes	RAIC	Imaging-defined post-radiation abnormalities, often considered synonymous with RICE in head and neck literature; may include contrast enhancement and/or T2/FLAIR hyperintensity.	6–24 months	Commonly described in skull base and head and neck proton therapy cohorts.
Pseudoprogression	PsP	Transient treatment-related increase in enhancement and/or edema mimicking tumor progression without true tumor growth.	<6 months (early)	Most commonly described in high-grade glioma after chemoradiotherapy; rare in benign skull base tumors.
Radiation Necrosis	RN	Irreversible radiation-induced tissue necrosis characterized by blood–brain barrier breakdown, mass effect, and potential neurological symptoms.	>12 months (may occur years later)	Late toxicity; may require corticosteroids, bevacizumab, or surgery.
Contrast-Enhancing Brain Lesion	CEBL	Purely descriptive radiologic term referring to an enhancing lesion of uncertain etiology (tumor vs radiation effect).	Not specific	Neutral imaging descriptor used prior to etiologic clarification.

**Table 2:** Quantitative summary of radiation-induced brain injury (RIBI) incidence and dose thresholds in key reference studies.

Study	Cohort Characteristics	Tumor Types	Modality	Endpoint Definition	Incidence Rate	Key Dosimetric and Clinical Findings
Lütgendorf-Caucig et al. (2024) [18]	N = 421 (Prospective)	CNS and Skull Base (Mixed)	Proton (PBS)	RICE (New contrast enhancement)	15% (Total) 4.5% (Symptomatic)	D1% > 57.6 Gy (RBE) to healthy CNS is a significant predictor (HR 3.73). Diabetes and prior RT are independent risk factors.
Engeseth et al. (2020) [20]	N = 127 (Retrospective)	Skull Base H&N (NPC, Sinonasal)	Proton (IMPT)	RAIC (Enhancement + T2 changes)	17.3% (Crude) 14.3% (3-yr Actuarial)	V67Gy (RBE) ≥ 0.17 cc associated with 63% risk of RAIC. 100% of lesions were asymptomatic; 45.5% resolved spontaneously.
Mattke et al. (2022) [46]	N = 147 (Prospective)	Skull Base Chordoma	Proton vs. Carbon Ion	Necrosis and Reaction	13.9% (Necrosis) 21.5% (Reaction)	Necrosis rates comparable between Protons and Carbon Ions. Risk correlates with Dmax and V70Gy volumes.
Brandes et al. (2008) [23]	N = 103 (Prospective)	Glioblastoma (GBM)	Photon + Temozolomide	Pseudoprogression	~30% (specifically in methylated MGMT)	Incidence driven by concurrent chemotherapy and tumor biology; Distinct from standard skull base radiation effects.

RICE: Radiation-induced contrast enhancement, RAIC: Radiation-associated image change.

**Table 3:** Skull base tumor series for each type of radiation-induced brain injury after radiotherapy.

Lesion Type	Tumor Population	Radiotherapy Modality	Cohort Size (*n*)	Incidence (%)	Key Dose–Volume Predictor(s)	Median Latency/Follow-Up	Reference
Pseudoprogression	Skull base tumors (mixed CNS/skull base prospective cohort)	Proton therapy	104	Early transient enhancement reported; lower than RICE rates (exact incidence variably reported)	Higher focal dose; concurrent systemic therapy	Within 3–6 months	Lütgendorf-Caucig et al., 2024 [18]
RICE	Head and neck skull base cancers	Proton therapy	127	17.3% overall; 3-year actuarial rate 14.3%	V70 GyRBE ≥ 0.17 cc strongly predictive (~63% RAIC when exceeded)	Median 21 months	Engeseth et al., 2020 [20]
Symptomatic RICE	Mixed CNS and skull base tumors (prospective)	Proton therapy	104	15% overall; 4.5% symptomatic	D1% CNS ≥ 57.6 GyRBE independently predictive	Median onset 11.8 months	Lütgendorf-Caucig et al., 2024 [18]
Temporal Lobe Reaction (subclinical + clinical)	Skull base chordoma and chondrosarcoma	Proton ± carbon ion therapy	147	21.5% temporal lobe reaction	Higher Dmax; V60–70 GyRBE; larger irradiated volume	Median 20 months	Mattke et al., 2022 [46]
Radiation Necrosis (Proton-dominant cohort)	Skull base chordoma and chondrosarcoma	Proton ± carbon ion therapy	147	13.9% necrosis	Higher Dmax; V70 GyRBE; older age	Median 20 months	Mattke et al., 2022 [46]
Radiation Necrosis (Carbon Ion Therapy)	Head and neck and skull base tumors	Carbon ion therapy	95	Up to 64%	Higher biological dose (RBE effect)	Variable	Miyawaki et al., 2009 [26]
Temporal Lobe Necrosis (Photon IMRT era)	Nasopharyngeal carcinoma involving skull base	Photon IMRT	616	2–5%	Total dose >70 Gy; temporal lobe high-dose exposure	2–5 years	Peng et al., 2012 [47]; Zhou et al., 2013 [48]

*n*: number of patients included in the study, RICE: Radiation-induced contrast enhancement, RAIC: Radiation-associated image change, Dmax: Maximum point dose. V70 GyRBE: Volume receiving ≥70 Gy (relative biological effectiveness), D1% CNS: Dose to hottest 1% of central nervous system.

**Table 4:** Anatomy-based distribution of post-radiotherapy imaging changes and common pitfalls in adult skull base irradiation.

Skull Base Target/Subsite	Adjacent CNS Structures at Risk	Typical Distribution of RICE/RAIC or Necrosis	Common Imaging Pitfall	Planning/Modality Notes
Clivus/petroclival region (e.g., chordoma, chondrosarcoma)	Medial temporal lobes; brainstem; cranial nerves VI–XII	Inferomedial/anterior temporal lobe (often ipsilateral to highest dose); occasionally brainstem	Focal temporal lobe enhancement may mimic progression in adjacent surgical bed; consider time course and dose distribution	High prescriptions (≈70–74 Gy [RBE]) increase risk; avoid systematic distal edge placement in temporal lobe; robust optimization and verification imaging in PBT
Cavernous sinus/Meckel’s cave (e.g., meningioma, perineural ACC)	Anterior temporal lobe; frontal lobe; optic pathway; cranial nerves III–VI	Anterior temporal lobe and/or inferior frontal lobe adjacent to cavernous sinus high-dose region	Perineural tumor spread vs. treatment-related cranial nerve/meningeal enhancement; correlate with pre-RT extent and serial evolution	In PBT, consider LET hotspots at end-of-range near temporal pole; distribute distal fall-off across beams when feasible
Sellar/suprasellar region (pituitary adenoma, craniopharyngioma)	Optic nerves/chiasm; hypothalamus; frontal lobes	Optic apparatus enhancement; less commonly adjacent frontal lobe changes	Optic nerve or chiasmal enhancement can mimic recurrence or compressive change; temporal lobe injury is uncommon at conventional doses unless re-irradiation	Typically lower dose (≈45–54 Gy) reduces necrosis risk; in re-irradiation, prioritize optic pathway constraints and avoid focal hotspots
Sinonasal cavity/paranasal sinuses with skull base extension	Inferior frontal lobes; temporal poles; olfactory groove	Inferior frontal lobe and anterior temporal poles depending on target and beam arrangement	Post-operative/inflammatory sinonasal enhancement vs. residual tumor; cortical enhancement may represent RICE rather than intracranial extension	For anterior proton beams, variable sinus filling can shift range; robust optimization and adaptive assessment are important
Nasopharynx/skull base invasion (UCNT/nasopharyngeal carcinoma)	Temporal lobes (inferomedial); brainstem	Inferomedial temporal lobes (often bilateral in large fields)	Temporal lobe necrosis vs. tumor recurrence at skull base; PET and perfusion MRI may help when MRI is equivocal	Historically higher risk with photon techniques; IMRT and PBT reduce high-dose volumes but hotspots remain relevant
Jugular foramen/petrous temporal bone	Cerebellum; lower cranial nerves; brainstem	Cerebellar hemispheres or pontomedullary region depending on target	Enhancement along lower cranial nerves vs. post-treatment change; assess diffusion/perfusion and clinical correlation	Beam paths near posterior fossa warrant strict brainstem constraints; consider artifact from hardware/skull base air–bone interfaces

## 4. Discussion

This review synthesizes current knowledge on MRI and PET alterations after proton and photon radiotherapy for adult skull base tumors. Radiation-induced brain injury results from endothelial damage and inflammatory cascades leading to blood–brain barrier breakdown and necrosis [46]. MRI alterations manifest as pseudoprogression, RICE, and radiation necrosis, forming a continuum of injury. Pseudoprogression occurs early and often resolves spontaneously [47]. RICE lesions appear months later and may regress; many remain asymptomatic [20]. Radiation necrosis is a severe late effect, presenting with ring enhancement and requiring intervention in symptomatic cases [40].

Proton therapy reduces high-dose exposure to the temporal and frontal lobes compared with photons, resulting in a lower incidence of necrosis. However, RICE remains common (15–17%) and typically resolves without treatment [18]. Carbon-ion therapy offers greater biological effectiveness but has higher necrosis rates (up to 64%) [26]. In chordoma and chondrosarcoma, temporal lobe reactions occur in ~22% and necrosis in ~14%, with risk strongly correlated with Dmax, V70, and patient age. Photon therapy for nasopharyngeal carcinoma and adenoid cystic carcinoma is associated with greater temporal lobe necrosis due to larger high-dose volumes, though IMRT has lowered rates [48,49]

Risk factors across modalities include high doses to small brain volumes, prior irradiation, older age, diabetes, and hypertension [50].

### 4.1. Clinical Implications

The management of post-radiation imaging changes requires a nuanced balance between oncologic vigilance and the avoidance of iatrogenic harm. Misinterpreting benign RICE as tumor progression can lead to disastrous consequences, including unnecessary salvage surgery or the premature cessation of effective therapy.

Studies show that at initial RICE occurrence, 39% of cases were misinterpreted as tumor progression. When RICE is incorrectly diagnosed as progressive disease, patients may undergo unnecessary surgical interventions or biopsy procedures to confirm what is actually a benign treatment effect. This exposes patients to surgical risks without therapeutic benefit [51]. Many RICE lesions resolve spontaneously; thus, short-interval MRI follow-up (3–6 months) is recommended. Advanced MRI and PET improve differentiation between necrosis and recurrence. In uncertain cases, combining multiple modalities (e.g., DWI, PWI, MRS, and FET-PET) increases diagnostic confidence.

The quantitative constraints identified in this review specifically limiting the temporal lobe volume receiving ≥ 67 Gy (RBE) to less than 0.17 cc provide a valuable reference point for treatment planning. However, these values should be interpreted as guidelines rather than absolute rules. In cases of extensive skull base chordomas/chondrosarcomas wrapping around the brainstem or temporal lobe, achieving these constraints may compromise tumor coverage. In such scenarios, the clinical team must weigh the risk of potentially reversible temporal lobe necrosis against the risk of fatal local tumor recurrence [52]. Furthermore, the biological uncertainty of the proton Bragg peak distal edge warrants careful planning. Because LET increases at the track end, treatment fields should ideally be arranged (using multiple beam angles) so that the distal edge does not terminate consistently within the same volume of the temporal lobe, thereby “smearing” the high-LET component and potentially mitigating the risk of focal injury [33].

Diabetes, hypertension, and previous irradiation should be considered when balancing tumor control and toxicity. When re-irradiation is needed, proton therapy is preferred to minimize additional brain dose.

### 4.2. Limitations of Current Evidence

Most data derive from retrospective series with small sample sizes, heterogeneous tumor types, and variable follow-up. Definitions of RICE, CEBL, and necrosis differ across studies, limiting comparability. Many series include both adult and pediatric patients or combine skull base and non-skull base tumors. Dosimetric parameters often vary because of differences in planning systems and RBE assumptions. Information on non-proton modalities (e.g., carbon ions) is scarce. Furthermore, many advanced MRI and PET studies focus on gliomas; direct application to skull base tumors requires caution due to anatomical constraints and differences in tumor biology.

### 4.3. Future Research

Future research must move beyond retrospective reporting. The integration of radiomics and artificial intelligence holds promise for identifying “radiomic signatures” of necrosis before they become clinically apparent [53,54,55]. Additionally, the standardization of terminology (as proposed by the EPTN) is a prerequisite for the success of multi-institutional registries. By speaking the same language distinguishing RICE from PsP and necrosis the oncology community can generate the high-quality data needed to refine dose constraints and improve survivor quality of life. This diagnostic challenge is even more critical in pediatric tumors such as diffuse intrinsic pontine glioma (DIPG), where imaging interpretation directly impacts therapeutic strategies, including re-irradiation, and where advanced MRI and metabolic PET play an essential role.

## 5. Conclusions

Radiation-induced brain injury remains a key late effect in skull base tumor radiotherapy, representing a spectrum from pseudoprogression to necrosis. Advanced MRI and PET imaging aid the differentiation from recurrence, while future work should harmonize definitions, integrate radiomics, and optimize dose constraints to reduce toxicity. AI and radiomics may enable the future detection of RICE at a very early stage and the initiation of preventive treatment to minimize the progression to radiation necrosis.

## Data Availability

No new data were created or analyzed in this study.

## Abbreviations

The following abbreviations are used in this manuscript: MRI, magnetic resonance imaging; PET, positron emission tomography; RT, radiotherapy; PBT, proton beam therapy; IMRT, intensity-modulated radiotherapy; VMAT, volumetric-modulated arc therapy; RIBI, radiation-induced brain injury; RICE, radiation-induced contrast enhancement; RAIC, radiation-associated image changes; CEBL, contrast-enhancing brain lesions; RBE, relative biological effectiveness; Gy, gray; GyRBE, gray (relative biological effectiveness); CNS, central nervous system; OAR, organ at risk; DWI, diffusion-weighted imaging; PWI, perfusion-weighted imaging; MRS, magnetic resonance spectroscopy; FDG, fluorodeoxyglucose; FET, fluoroethyl-L-tyrosine.
